# Interactive Health Technology Tool for Kidney Living Donor Assessment to Standardize the Informed Consent Process: Usability and Qualitative Content Analysis

**DOI:** 10.2196/47785

**Published:** 2024-07-09

**Authors:** Fernanda Ortiz, Juulia Grasberger, Agneta Ekstrand, Ilkka Helanterä, Guido Giunti

**Affiliations:** 1 Abdominal Center–Nephrology Helsinki University Hospital Helsinki Finland; 2 Faculty of Medicine Helsinki University Helsinki Finland; 3 Abdominal Center–Transplantation and Liver Surgery Helsinki University Hospital Helsinki Finland; 4 Faculty of Medicine University of Oulu Oulu Finland; 5 School of Medicine Trinity College Dublin Ireland

**Keywords:** eHealth, kidney living donor, informed consent, telemedicine, process standardization, kidney, donor, tool, usability, psychological impact, utility, smartphone, coping, surgery

## Abstract

**Background:**

Kidney living donation carries risks, yet standardized information provision regarding nephrectomy risks and psychological impacts for candidates remains lacking.

**Objective:**

This study assesses the benefit of interactive health technology in improving the informed consent process for kidney living donation.

**Methods:**

The Kidney Hub institutional open portal offers comprehensive information on kidney disease and donation. Individuals willing to start the kidney living donation process at Helsinki University Hospital (January 2019-January 2022) were invited to use the patient-tailored digital care path (Living Donor Digital Care Path) included in the Kidney Hub. This platform provides detailed donation process information and facilitates communication between health care professionals and patients. eHealth literacy was evaluated via the eHealth Literacy Scale (eHEALS), usability with the System Usability Scale (SUS), and system utility through Likert-scale surveys with scores of 1-5. Qualitative content analysis addressed an open-ended question.

**Results:**

The Kidney Hub portal received over 8000 monthly visits, including to its sections on donation benefits (n=1629 views) and impact on donors’ lives (n=4850 views). Of 127 living kidney donation candidates, 7 did not use Living Donor Digital Care Path. Users’ ages ranged from 20 to 79 years, and they exchanged over 3500 messages. A total of 74 living donor candidates participated in the survey. Female candidates more commonly searched the internet about kidney donation (n=79 female candidates vs n=48 male candidates; *P*=.04). The mean eHEALS score correlated with internet use for health decisions (*r*=0.45; *P*<.001) and its importance (*r*=0.40; *P*=.01). Participants found that the Living Donor Digital Care Path was technically satisfactory (mean SUS score 4.4, SD 0.54) and useful but not pivotal in donation decision-making. Concerns focused on postsurgery coping for donors and recipients.

**Conclusions:**

Telemedicine effectively educates living kidney donor candidates on the donation process. The Living Donor Digital Care Path serves as a valuable eHealth tool, aiding clinicians in standardizing steps toward informed consent.

**Trial Registration:**

ClinicalTrials.gov NCT04791670; https://clinicaltrials.gov/study/NCT04791670

**International Registered Report Identifier (IRRID):**

RR2-10.1136/bmjopen-2021-051166

## Introduction

The optimal choice for a patient awaiting a kidney transplant is to receive the organ from a living donor. Despite efforts to boost living kidney donation, global rates vary widely [1]. Kidney donation involves risks, making it crucial to ensure donor candidates receive the necessary information to make informed decisions. The process begins with a medical candidacy assessment and comprehensive details about the nephrectomy’s process and consequences. Health care professionals must confirm that living kidney donation candidates comprehend risks, understand potential outcomes for both donor and recipient, and can independently decide, leading to an informed consent document.

Informed consent procedures vary across countries, transplant centers, and among health care professionals, especially surgeons and nephrologists [2,3]. Standardizing information provided to living kidney donation candidates is essential [4]. There is agreement on communicating all potential health, economic, and psychosocial risks to living donors. Various guidelines outline matters to disclose, but their implementation varies by the transplant center. A recent study in the Netherlands found variation in basic procedure knowledge among potential living kidney donation candidates even though the information followed guidelines. A possible cause was donors receiving information in referring hospitals rather than transplant centers [5]. A survey in Europe highlighted gaps in discussions between health care professionals and potential kidney donors about long-term risks [6].

Health care technology has undergone significant changes, going beyond digitizing health records. Digital transformation implies broad technology use, incorporating electronic health record data and enabling telemedicine. A 2019 US survey on digital services for living donor candidates revealed the potential for mobile health to enhance donor follow-up and aid centers in meeting reporting thresholds. Concerns about cybersecurity, usability, and cost-effectiveness were raised [7]. A 2022 survey in the United States supported telemedicine’s convenience for improving access to and coordination of living donor evaluation. However, participants expressed less confidence in removing regulatory office barriers. Pilot studies focusing on living donor candidate education with eHealth tools show promising results [8-10].

**Figure 1:**
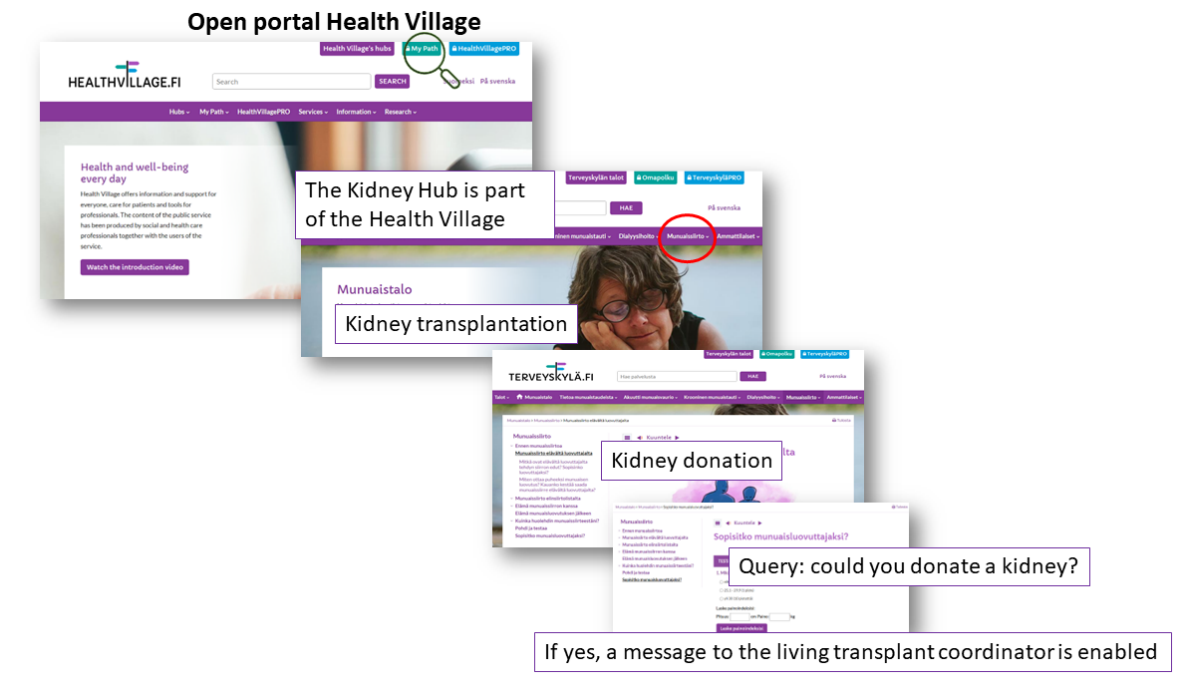
The digital process for providing information to kidney donors on the hospital website.

**Figure 2:**
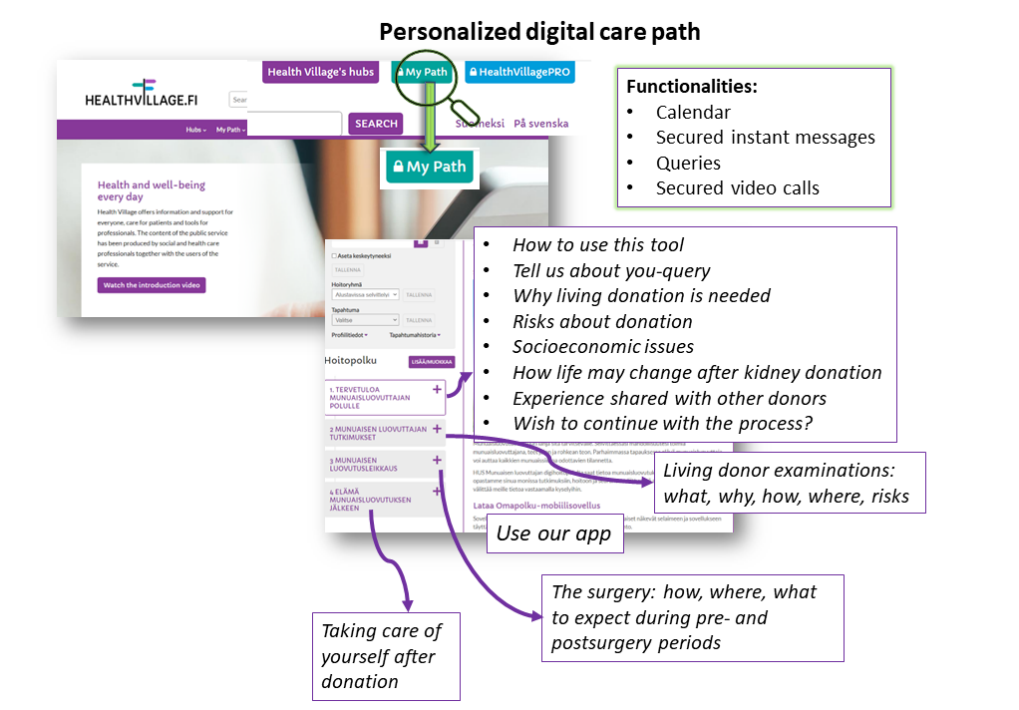
Description of the personalized information steps included in Living Donor Digital Care Path.

Finland’s Ministry of Social Affairs and Health partially funded the *National Action Plan on Organ Donation and Transplantation* to promote online health information and eHealth tools. The establishment of the Virtual Hospital led to the “Health Village,” providing information, patient care, and professional tools. The Health Village, developed by Finnish university hospitals, includes specific hubs like the Kidney Hub for individuals with kidney disease [11]. In December 2018, Helsinki University Hospital launched the Living Kidney Donor Digital Care Path (LD-dcp) in the Kidney Hub. LD-dcp, exclusively available to those considering kidney donation, aims to increase the number of donors by offering standardized information, secure messaging pathways, and teleconsultation options. Figure 1 illustrates our institution’s process for providing digital information to candidates for living kidney donation. LD-dcp initially focuses on active use for education and continues on to telemedicine and messaging throughout the kidney donation evaluation process. Figure 2 outlines the content of LD-dcp.

So far, the performance and usefulness of these eHealth solutions have not been studied. We aimed to examine the benefit and usability of eHealth services designed for kidney donor candidates as a tool for a standardized informed consent process. The secondary aims were to investigate living donor candidates’ eHealth literacy and patterns of use of digital health services concerning kidney donation.

## Methods

### Participants

This was a prospective cross-sectional survey study involving all LD-dcp users (N=127) from January 2019 to December 2021 evaluated at the Helsinki University Hospital Department of Nephrology. We approached the participants by phone, text messaging, and/or email, considering the participants’ preferences. Detailed information on the study protocol has been previously published [12]. Briefly, we used 3 questionnaires, and the answers were provided electronically. The first one gathered information about sociodemographic factors, device ownership, and purpose of use. The second assessed eHealth literacy with the eHealth Literacy Scale (eHEALS), an 8-item Likert scale that measures perceived skills at finding, evaluating, and applying electronic health information to health problems (1=strongly disagree to 5=strongly agree). The scale is based on a model that distinguishes between 6 types of literacy skills: traditional literacy, health literacy, information literacy, scientific literacy, computer literacy, and media literacy. The third questionnaire was intended to assess the Kidney Hub and LD-dcp platform’s ease of use by applying the System Usability Scale (SUS), and users’ feedback on LD-dcp was explored with 5-point Likert scale questions (1=strongly disagree to 5=strongly agree) and 1 open question for qualitative analysis (“Is there anything you wish kidney donors should be warned about that you were not?”). Written informed consent was obtained electronically within LD-dcp (Multimedia Appendix 1).

Individuals willing to donate a kidney seek information from different sources, one of them being the Kidney Hub. Therefore, access to the Kidney Hub was included in the analysis to serve as a reference for the use of this open-access portal in the context of kidney transplantation and living kidney donation.

### Statistical Analysis

Webpage demographics and use patterns of the open-portal Kidney Hub were analyzed with Google Analytics and Power BI (Microsoft Corp). This study is descriptive, and sample size calculation was not needed because all LD-dcp users were invited to participate. Descriptive statistics were used to summarize participants’ backgrounds and characteristics. Categorical variables were presented as absolute and relative frequencies. Continuous variables were presented as mean and SD or median and IQR depending on the distribution. A *P* value of less than 5% was considered statistically significant. For eHEALS and SUS, quantitative analysis followed the instruments’ scoring system and the 5-point Likert-item response. The Cronbach α correlation was calculated to assess internal consistency. The Pearson correlation, Mann-Whitney *U*, and *χ*^2^ tests were used when appropriate. Qualitative content analysis was used on the open-ended question. We evaluated the presence of words and concepts within the data (meaning units), synthesized them in code units, and made a further analysis by counting the frequencies of the detected categories.

### Ethical Considerations

The research protocol has been approved by the Helsinki University Hospital ethical committee (HUS/501/2021) to ensure that the work is done in accordance with the Declaration of Helsinki and the Declaration of Istanbul. This clinical trial has been registered in ClinicalTrials.gov (NCT04791670). The consent to participate in this survey was carried on electronically and integrated and secured into LD-dcp.

## Results

**Figure 3:**
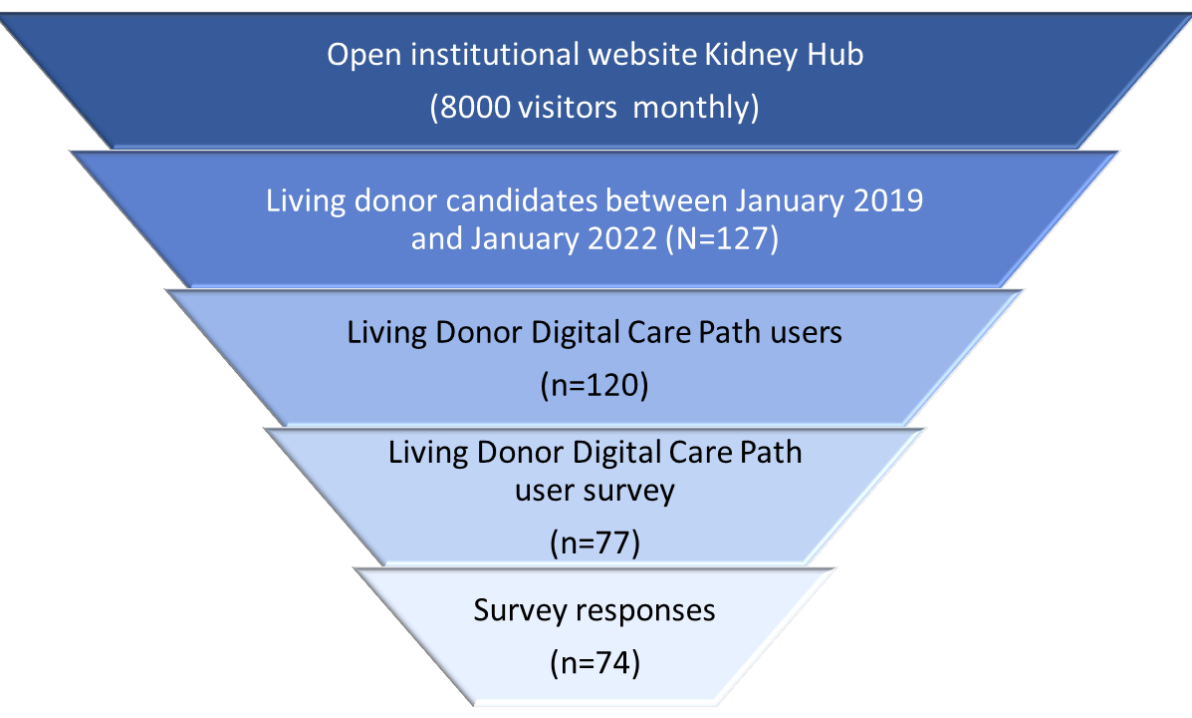
Flowchart of the users of the digital services provided by the Kidney Hub.

The results of the use of the institutional open portal Kidney Hub are presented, followed by the use of LD-dcp, and finally the results of the survey of LD-dcp users to evaluate their experience with the digital services. A flowchart of users of the digital services is depicted in Figure 3.

### Use of the Kidney Hub Open Portal

Initially, we analyzed the use of the Kidney Hub open portal, considering that it would serve as an initial information source for patients needing a kidney transplant and for potential kidney donors. The Kidney Hub was accessed between January 2019 and December 2021 on average by 8000 (SD 2217) visitors per month, of whom 5680 (71%) were female. Altogether, 4800 of 8000 (60%) of the Kidney Hub visitors were from Finland, 2480 (31%) from Sweden, 480 (6%) from the United States, and less than 80 (1%) from Norway, Germany, Spain, the United Kingdom, Bulgaria, Russia, and Canada. It was most frequently visited between midday and 10 PM on weekdays. In all, 2000 (25%) of the 8000 visitors were older than 65 years, 1680 (21%) were aged 25 to 34 years; 1520 (19%) were aged 55 to 64 years, 1120 (14%) of the visitors were aged 35 to 54 years, and only 640 (8%) visitors were aged less than 25 years. The website was accessed from a mobile phone by 4800 of 8000 users (60%); 2480 (31%) accessed the website from a desktop and 640 (8%) from a tablet.

The Kidney Hub includes tips for patients on how to initiate a conversation about living kidney donation. This page was visited over 2600 times. The page on general information about the benefits of living kidney donation was visited 1629 times, and the page on how living kidney donation impacts the donor’s life was visited 4850 times.

### Use of LD-dcp

All donor candidates were invited to join LD-dcp from January 2019 onwards. This service is only open to those who express their willingness to donate a kidney to the living donor transplant coordinator. This service is free of charge to the living donor candidates and only requires internet access. A reminder is sent to those who do not activate the system within 1 week. Altogether, 127 living donor candidates initiated the evaluation process, of whom 79 (62%) were female. Six donor candidates preferred not to use digital services and 1 had language barriers. A total of 48 of 120 LD-dcp users actively interacted with this system at 6 months from activation. The age of the LD-dcp users ranged from 20 to 79 years, and 91 (72%) of them were aged between 40 and 69 years. In all, 3511 messages were exchanged through the LD-dcp, of which 2247 (64%) were from female users. A total of 30% of the participants (n=22) had already donated a kidney when they answered the survey, and some participants did not donate, meaning that they did not experience the entire process. The quality analysis brought to light 5 different topics: unexpected discontinuation of the donor evaluation process due to unknown medical conditions, with consequent disappointment; surprise about extensive, time-consuming medical evaluation and lab tests; practical issues related to the stay on the ward and discharge after surgery; impact of kidney transplantation on both donor and recipient, focused on expectations; and finally a positive message to others considering donation (“Life doesn’t change that much”).

### Survey Study Participants

Of all 120 LD-dcp users invited to participate in this study by a message through LD-dcp, 77 agreed. The surveys were answered by 74 participants (for a response rate of 58%) and by then, 23% (n=31) had already donated a kidney. The sociodemographic data of the participants are shown in Table 1.

**Table 1:** Sociodemographic data from 74 Living Donor Digital Care Path users who responded to the questionnaire.

Sociodemographic data		Values
Age (years), mean (SD)		50.3 (13.45)
Age range (years)		23-76
Sex (female), n (%)		45 (61)
**Education, n (%)**		
	N/A^a^	15 (20)
	Primary school	3 (4)
	High school or vocational school	23 (31)
	Polytechnic or university	31 (42)
	Other	2 (3)
**Working status, n (%)**		
	N/A	15 (20)
	Student	1 (1)
	Unemployed	1 (1)
	Employed	45 (61)
	Retired	2 (3)
	Other	10 (14)
**Annual income category^b^, n (%)**		
	N/A	15 (20)
	Don’t want to answer	2 (3)
	Under €20,000	4 (5)
	€20,000-€40,000	21 (28)
	€40,000-€60,000	18 (24)
	Over €60,000	14 (19)
**Live alone, n (%)**		
	N/A	15 (20)
	Yes	7 (10)
	No	52 (70)

^a^N/A: not available.

^b^The exchange rate at the time of writing was US $1.00=€0.93.

### Living Donor Candidates’ Patterns of Use of Digital Technology

A total of 15 of 74 participants did not respond to this query (for a response rate of 80%). All 59 respondents had a smartphone; 4 (5%) did not have a computer and 26 (35%) did not have a tablet. A total of 52 of 59 respondents (88%) used their smartphones for sending messages (including instant messaging), navigating the internet, taking photos or videos, and reading emails several days a week or on an everyday basis. A total of 10 of 59 respondents (17%) never watched TV or movies on their smartphone. Social media was used by 42 of 59 respondents (71%), health apps by 41 (69%); the participants used these services several days a week or every day. A total of 27 of 59 respondents (46%) never used their smartphone for gaming.

### Use of the Internet for Searching for Information About Health

**Figure 4:**
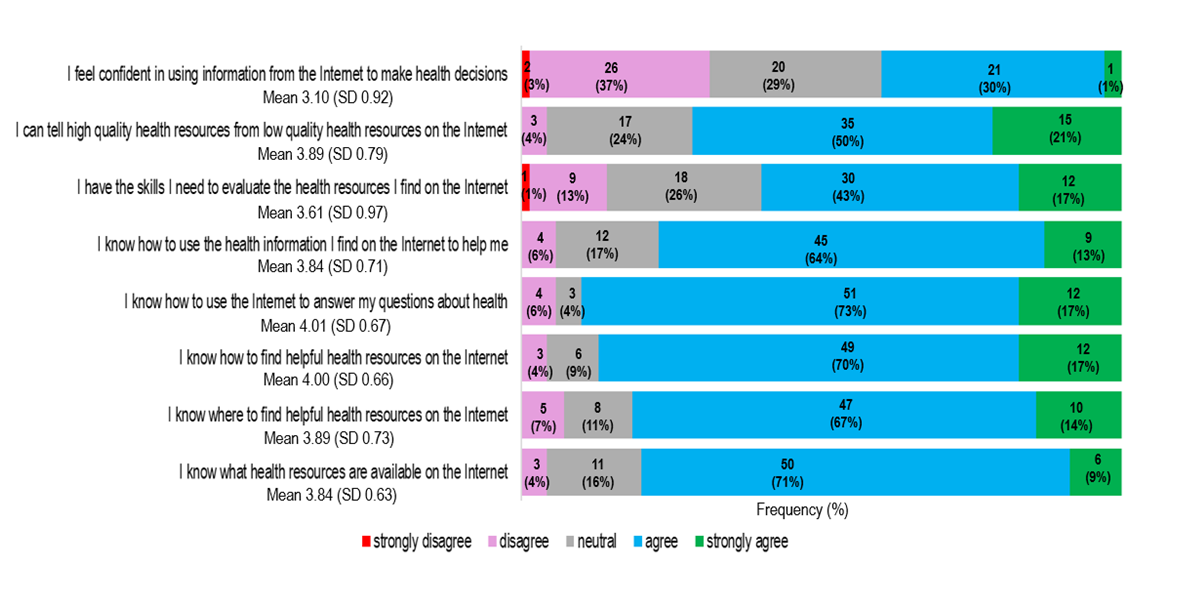
Scores for the eHealth Literacy Scale (eHEALS). The frequency of responses is expressed in percentages in each bar (n=74). The mean value was calculated from answers on a 1 to 5 Likert scale (1=strongly disagree; 2=disagree; 3=neutral; 4=agree; 5=strongly agree).

Over 86% of the respondents (64/74) agreed or strongly agreed that the internet was important and helpful to find out about health issues. Almost 80% of the participants (59/74) knew what, where, and how to find health resources on the internet. However, fewer participants felt they had the skills to evaluate the quality of the information they found, and only 30 of 74 (40%) felt confident in using this information to make decisions about their health. The detailed results of the eHEALS survey are displayed in Figure 4. The Cronbach α was 0.86, indicating good internal consistency. The mean eHEALS score was 3.77 (IQR 3.5-4.0). Cutoff points have not been validated for the eHEALS, and scores cannot be categorized reliably. There was no significant correlation between eHEALS score and age (*r*=–.127; *P*=.30). Mean eHEALS score was not statistically different across educational level (*P*=.25), working status (*P*=.16), or income level (*P*=.29). Mean eHEALS score was similar between men and women (*P*=.34) and positively correlated with using the internet for decision-making (*r*=0.45; *P*<.001) about health issues and agreeing on the importance of using the internet for health-related issues (*r*=0.40; *P*=.01).

### Usefulness and Satisfaction With LD-dcp

A total of 54 of 74 participants (73%) had searched the internet for information about living kidney donation before contacting the transplant coordinator. Searching on the internet about kidney donation was significantly more common among women (46/54, 84% women vs 28/54, 52% men; *P*=.04). Of the 52 participants who properly completed the questionnaire about socioeconomic status (52 out of 74, 70.3%), searching on the internet about kidney donation was not related to education level (2/2, 100% of those with primary education; 12/18, 68% of those with high school education; and n=21/26, 81% of those with university education; other 3/6, 50%, *P*=.48), working status (1/1, 100% of students; 30/38, 79% of employed people; 1/1, 100% of retired people; and other 6/12, 50%; *P*=.33) or income (2/3, 67% of those with income below €20,000/year; 10/15, 67% of those with income between €20,000 and €40,000/year; 12/16, 75% of those with income between €40,000 and €60,000/year; 10/12, 86% of those with income over €60,000/year; and don't want to tell n=4/6, 67%; *P*=.72 [The exchange rate at the time of writing was US $1.00=€0.93]). The sources of information about living kidney donation included the hospital website and the Kidney Hub (24/74, 33%), patient associations (30/74, 40%), general search engines (ie, Google, Wikipedia, and social media; 17/74, 23%), and other donors (4/74, 5%). A total of 66 of 74 participants (90%) considered that the information about living kidney donation available in the Kidney Hub open portal was useful.

**Figure 5:**
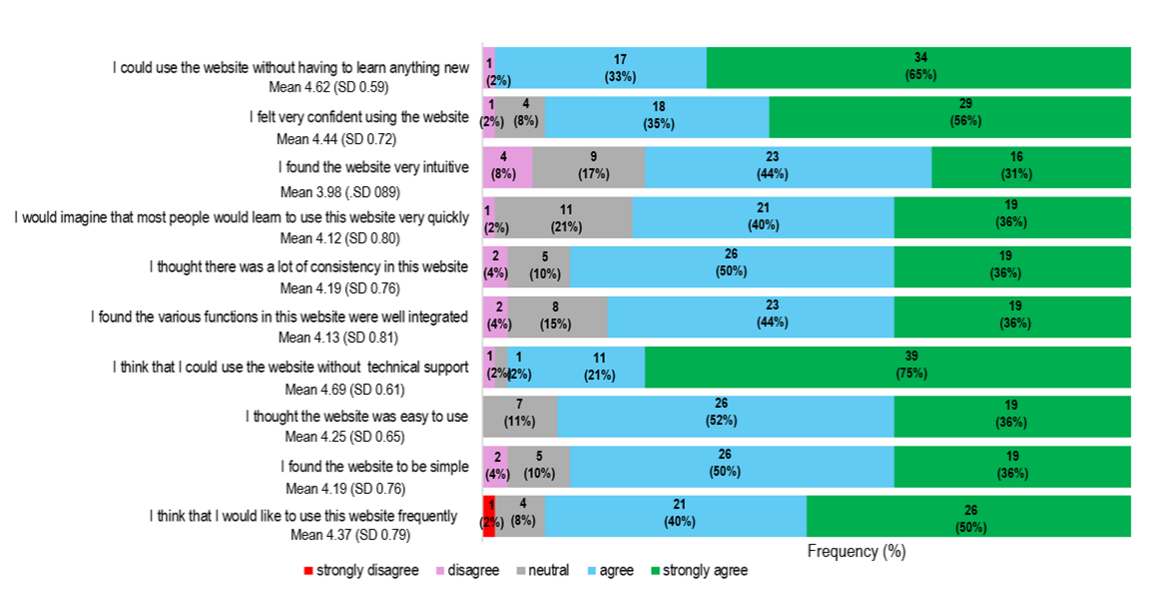
Patients’ experience with Living Kidney Donor Digital Care Path (n=52). The technical usability was assessed with the System Usability Scale (SUS). The mean value was calculated from answers on a 1 to 5 Likert scale (1=strongly disagree; 2=disagree; 3=neutral; 4=agree; 5=strongly agree).

**Figure 6:**
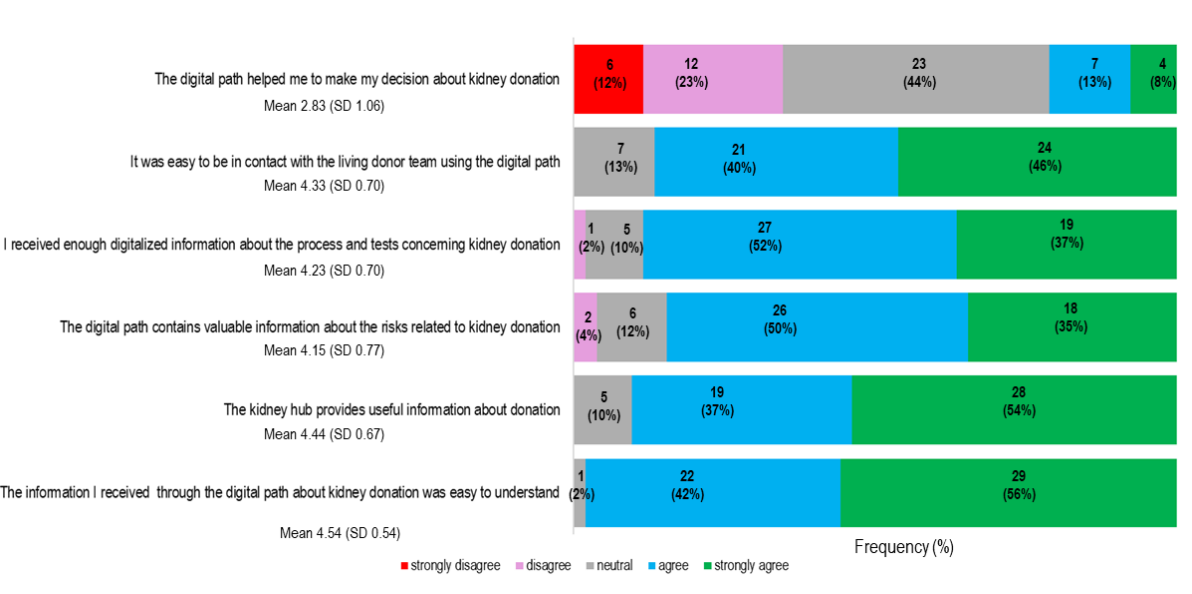
Patients’ experience with Living Kidney Donor Digital Care Path (n=52). The utility of the content was calculated as the mean of answers on a 1 to 5 Likert scale (1=strongly disagree; 2=disagree; 3=neutral; 4=agree; 5=strongly agree).

The results of the SUS and Utility Scale queries are shown in Figures 5 and 6, respectively. The Cronbach α of the SUS was 0.89, implying good reliability, and the Cronbach α of the Utility Scale was 0.73, indicating acceptable reliability. We were unable to detect any gender differences in the SUS mean score (men: mean score 4.2, SD .63; women: mean score 4.3, SD .48; *P*=.35) or the Utility Scale mean score (men: mean score 4.1, SD .51; women: mean score 4.1, SD .49; *P*=.95). Neither score correlated with age (*r*=–0.127; *P*=.91 and *r*=0.016; *P*=.96, respectively). The participants agreed that the information about kidney donation available in the Kidney Hub is useful (mean score 4.44, SD .67).

The last question in the survey allowed the participants to provide free feedback about any missing advice during the kidney donation process (“Is there anything you wish kidney donors should be warned about that you were not?”). The most common issues of concern are detailed in Table 2.

**Table 2:** Quality analysis of the answers to the open question “Is there anything you wish kidney donors should be warned about that you were not?”

Content	Description	Quotes
More information	Detailed information about the evaluation process itself	“It would have been good to know early during the process what tests will be done, and for what reason.” “The number of blood tests was surprisingly high.”
Own health	Health status during the evaluation process	“I thought I was healthy before starting the process, but some risks came up. It was anyway good to know.”
Surgery	Practical issues about postoperative recovery	“It’s needed to organize beforehand how are you going to manage your daily life after discharge from surgery, particularly when both donor and recipient live under the same roof.” “I would advise others about everything happening in the ward after the surgery, to prepare yourself.”
Expectations	The kidney recipient’s well-being after surgery	“Kidney recipient might recover slowly and have complications from his own disease or cannot tolerate well immunosuppression. This is a burden for both the donor and recipient.”
Long term effects	About coping after donation	“Make clear to others that life doesn’t change much after donation.”

## Discussion

We found that Finnish living kidney donor candidates actively used the internet for health-related issues and felt confident about the acquired information. Women were more engaged in this activity, and the dedicated open portal section regarding the impact of living kidney donation on the donor’s life was highly visited. Interestingly, almost half of male donor candidates did not initiate an internet search before commencing the organ donation process. Contrary to general internet use in Finnish society, where men are more active and only 67% of those older than 65 years use it monthly [13], there is concern that almost 30% of individuals older than 65 years are not receiving kidney donation information. Therefore, alternative media such as TV, magazines, or personal interactions are necessary for this age group. Active internet searches by living kidney donor candidates explained why LD-dcp did not influence decision-making. The primary information source was the web portal from the patients’ association, emphasizing coordinated efforts. Using written information and checklists could standardize the process, as suggested by a survey from the European Renal Association and the European Society of Organ Transplantation [6].

Technological advancements, especially during the COVID-19 pandemic, saw increased reliance on telemedicine and web-based information by living kidney donor candidates. However, a Dutch study revealed that delivery modes mostly focused on individual and passive learning, lacking group learning or active knowledge construction [14]. Educational platforms like the iChoose Kidney Aid eHealth portal showed significant knowledge improvement but did not increase access to transplantation [15]. Although living kidney donor candidates found information from the Kidney Hub useful, the impact on long-term outcomes remains uncertain [16].

Interactive platforms like the Talking About Live Kidney Donation Social Worker Intervention and the Living Organ Video Educated Donors (LOVED) program showed promise but faced implementation challenges due to technology concerns [9,10]. In the liver donation process, the Evaluation of Donor Informed Consent Tool (EDICT) displayed initial positive outcomes [17]. Digital technologies offer opportunities to enhance processes to obtain consent. For instance, our research obtained electronic consent through LD-dcp, an approach that has been similarly explored in cancer research, although it met with some resistance [18].

Our survey’s open question allowed LD-dcp users to share their experiences, although the tool’s short period of use limited long-term effect assessments and excluded many participants without kidney donation experience. A larger study indicated living kidney donor concerns about surgery, kidney health, lifestyle changes, psychosocial impacts, and positive effects on donor-recipient relationships [19]. Evaluation experiences highlighted that living kidney donor candidates invest emotions and time and potentially face disappointment if contraindications for donation arise. This underscores the necessity for presurgery preparation and anxiety reduction, as well as support during evaluation, minimizing unnecessary delays [20].

Limitations include LD-dcp availability only in Finnish, potential bias toward positive eHealth experiences due to nonparticipation, and a scope limited to Helsinki. Nonetheless, our findings showcase living kidney donor candidates benefiting from LD-dcp, receiving standardized donation process information, and embracing digital services positively. However, its contribution to decision-making was limited, possibly due to prior active internet searches. Future eHealth services should integrate therapeutic education, self-management promotion, and seamless integration into electronic health records. Our future focus involves updating LD-dcp based on this study’s insights and expanding its nationwide use.

## Appendix

Multimedia Appendix 1 Questionnaires.

## Abbreviations

EDICT: Evaluation of Donor Informed Consent Tool
eHEALS: eHealth Literacy Scale
LD-dcp: Living Donor Digital Care Path
LOVED: Living Organ Video Educated Donors
SUS: System Usability Scale
