# Supplementary material for: Interactive Health Technology Tool for Kidney Living Donor Assessment to Standardize the Informed Consent Process: Usability and Qualitative Content Analysis
Source: JMIR Form Res. 2024 Jul 9;8:e47785. doi: 10.2196/47785 (PMC11267092; doi:10.2196/47785)
Supplement: Multimedia Appendix 1 [file formative_v8i1e47785_app1.pdf]

General information

Participant ID

1. Date of birth:
2. Gender:
3. Level of education:
   Primary Secondary Tertiary University Prefer not to answer
4. Income category (yearly):
   less than 20000€ 20000€ - 40000€ 40000€ - 60000€ over 60000€ Prefer not to answer
5. Working status:
   student unemployed working retired other
6. Do you live alone? yes - no
7. Do you own any of the following items? – Mark <u>all</u> that apply.
   a. Computer □
   b. Smart mobile phone □
   c. Tablet □
   d. None □
   e. Prefer not to answer □
8. If you own a mobile device (phone or tablet), please mark if you use it for any of the following. Mark <u>all</u> that apply.
   f. Send/Receive text messages □
   g. Instant messaging (Whatsapp, etc) □
   h. Send/Receive emails □
   i. Take photos or videos □
   j. Navigating in the Internet □
   k. Social Media (WhatsApp, Facebook, Twitter, Instagram, etc) □
   l. Use of health apps (Fitbit, Nike+, etc) □
   m. Use other apps □
   n. Prefer not to answer □
9. If you use the internet, how often do you use it?

	Every day	Several days a week	About once a week	Less often	Never
Read e-mails					
Search information (Google or similar)					
Read newspapers					
Watch movies or TV					
Social Media					
Play games					

eHealth Literacy Scale

We would like to ask you for your opinion and about your experience using the Internet for health information. For each statement, tell me which response best reflects your opinion and experience *right now*.

1. How **useful** do you feel the Internet is in helping you in making decisions about your health?

Not important at all | Not important | Unsure | Important | Very important

2. How **important** is it for you to be able to access health resources on the Internet?

Not important at all | Not important | Unsure | Important | Very important

3. I know **what** health resources are available on the Internet

Strongly disagree | Disagre | Undecided | Agree | Strongly Agree

4. I know **where** to find helpful health resources on the Internet

Strongly disagree | Disagre | Undecided | Agree | Strongly Agree

5. I know **how** to find helpful health resources on the Internet

Strongly disagree | Disagre | Undecided | Agree | Strongly Agree

6. I know **how to use** the Internet to answer my questions about health

Strongly disagree | Disagre | Undecided | Agree | Strongly Agree

7. I know how to use **the health information** I find on the Internet to help me

Strongly disagree | Disagre | Undecided | Agree | Strongly Agree

8. I have the skills I need to **evaluate** the health resources I find on the Internet

Strongly disagree | Disagre | Undecided | Agree | Strongly Agree

9. I can tell **high quality** health resources from **low quality** health resources on the Internet

Strongly disagree | Disagre | Undecided | Agree | Strongly Agree

10. I feel **confident** in using information from the Internet to make health decisions

Strongly disagree | Disagre | Undecided | Agree | Strongly Agree

Thank you!

Query about the Digital Care Path for Living Donors

Participant ID (researcher fulfills):

- Have you searched in the internet information about kidney donation before your first call to the nephrology outpatient clinic or transplant coordinator? Yes / No
- Have you already donated a kidney? Yes / No

Evaluate the next affirmations from a scale from 1 to 5

(1: strongly disagree, 2: disagree, 3: neutral, 4: agree, and 5: strongly agree)

Technical section (easy of use)

---

1. I think that I would like to use this website frequently ---------------------------------- ☐
2. I found the website to be simple -- ☐
3. I thought the website was easy to use -- ☐
4. I think that I could use the website without the support of a technical person ----- ☐
5. I found the various functions in this website were well integrated ------------------- ☐
6. I thought there was a lot of consistency in this website --------------------------------- ☐
7. I would imagine that most people would learn to use this website very quickly --- ☐
8. I found the website very intuitive --- ☐
9. I felt very confident using the website -- ☐
10. I could use the website without having to learn anything new ------------------------ ☐

Medical section (utility)

---

1. The kidney transplant section of the Kidney hub provided useful general information about kidney donation --- ☐
2. The information I received through the digital treatment path about kidney donation was easy to understand --- ☐
3. The digital treatment path contains valuable information about the risks related to kidney donation -- ☐
4. I received enough digitalized information about the process and tests concerning kidney donation --- ☐
5. It was easy to be in contact with the living donor team using the digital treatment path ---------- ☐
6. The digital treatment path helped me to make my decision about kidney donation --------------- ☐

Is there anything you wish kidney donors should be warned you about that you were not?
